# Comparison of mass spectrometry and flow cytometry in measuring minimal residual disease in multiple myeloma

**DOI:** 10.1002/cam4.4254

**Published:** 2021-09-08

**Authors:** David Foureau, Manisha Bhutani, Fei Guo, Katherine Rigby, Marina Leonidas, Elise Tjaden, Andee Fox, Shebli Atrash, Barry Paul, Peter M. Voorhees, Saad Z. Usmani

**Affiliations:** ^1^ Immune Monitoring Core Laboratory Levine Cancer Institute Atrium Health Charlotte North Carolina USA; ^2^ Department of Hematologic Oncology Levine Cancer Institute Atrium Health Charlotte North Carolina USA

**Keywords:** mass spectrometry, multiple myeloma, NGF‐MRD

## Abstract

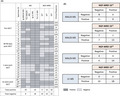

## CONFLICT OF INTEREST

D.F. received research funding from TeneoBio; M.B. served on speaker bureau for Amgen, BMS, and Takeda; consultant for Sanofi Genzyme; received research funding from Janssen, MedImmune, Takeda, and Prothena. D.P. and B.B are employees of TeneBio; B.P. was formally employed by Bristol‐Myers Squibb; S.A. received research funding from Mundipharma‐EDO, consulted for Celgene, served in advisory committee for Takeda, Celgene, Amgen, Sanofi, Karyopharm; P.V. served on speaker bureau of Celgene, Janssen, Takeda, served in advisory committee for Amgen, Celgene, Janssen; S.U. received research funding from Array BioPharma, Amgen. Celgene, Onyx, Sanofi, Janssen, Pharmacyclics, Bristol‐Myer‐Squibb, Seattle Genetics, SkylineDX, TeneoBio, served on speaker bureau for Amgen, Celgene, Takeda, Janssen, served in advisory committee for Abbvie, Amgen, Celgene, GSK, BMS, Sanofi, SkylineDX, Takeda, Seattle Genetics, Janssen.

## AUTHOR CONTRIBUTIONS

David Foureau and Manisha Bhutani designed the study, collected data, performed analyses, and wrote the paper. Fei Guo, Katherine Rigby, Marina Leonidas, Elise Tjaden, and Andee Fox provided lab support and collected data. Shebli Atrash, Barry Paul, Peter M. Voorhees, and Saad Z Usmani provided study patients and participated in study design. All authors reviewed the final draft of the paper.

To the Editor:

Complete response (CR), as defined by the International Myeloma Working Group criteria, represents a heterogeneous state that ranges from minimal residual disease (MRD)‐negative to MRD‐positive. The strong correlation between MRD status and survival in multiple myeloma (MM) has led to the adaption of techniques to identify MRD with great precision. Currently used techniques such as the standardized next‐generation flow cytometry (NGF) and next‐generation sequencing can detect rare malignant cells in bone marrow aspirates at ≤1 in 10^5^ cells. Based on the principle that myeloma cells produce antibodies with unique amino acid and mass spectra fingerprints, mass spectrometry (MS) may offer a non‐invasive approach to sequential MRD testing in peripheral blood. We compared the effectiveness of MS to NGF for capturing MRD status by analyzing the peripheral blood of 24 patients with MM by both techniques. All patients were enrolled in an IRB‐approved specimen collection protocol.

For each patient, serum M‐protein was measured at diagnosis (*n* = 17) or at relapse (*n* = 7) using standard serum protein electrophoresis (SPEP), immunofixation (IFE), and free light chain (FLC) immunoassays. For MS, serum immunoglobulins (IgG, IgA, IgM, total κ/λ, and free κ/λ light chains) were immune enriched using paramagnetic microparticles covalently coated with polyclonal sheep antibodies (The Binding Site Group Ltd). Mass spectra were acquired separately for each isotype‐specific immune‐enriched eluate. To detect intact immunoglobulins, we used matrix‐assisted laser desorption time‐of‐flight MS (MALDI‐TOF‐MS)^1^, ^2^, ^3^ with liquid chromatography and electrospray ionization time‐of‐flight MS (Ig‐LC‐MS). Similarly, to detect FLC, we used MALDI‐TOF‐MS with liquid chromatography and electrospray ionization time‐of‐flight MS (FLC‐LC‐MS). The minimum input for the assay was 250 μl; total time from sample preparation to results was 1 h for MALDI‐TOF plus 1 h for LC‐MS. We defined an M‐protein as the presence of a sharp peak distinguishable from the polyclonal background in the light chain mass‐to‐charge (*m*/*z*) range, with a peak‐height signal/noise value >3. In all patients, the M‐protein isotypes identified by MS were concordant with SPEP, IFE, and FLC results. We found that the most common type of M‐protein in our cohort was IgG (15/24 patients, 62.5%) followed by IgA (3/24 patients; 12.5%). Six patients had light chain only MM. While FLCκ only was detected on IFE in five patients, an additional M‐protein was detected by MS: IgGκ (*n* = 4) or IgGλ/FLCλ/FLCκ (*n* = 1). Two of these patients also had glycosylated monoclonal light chains detected by MS.

**FIGURE 1:**
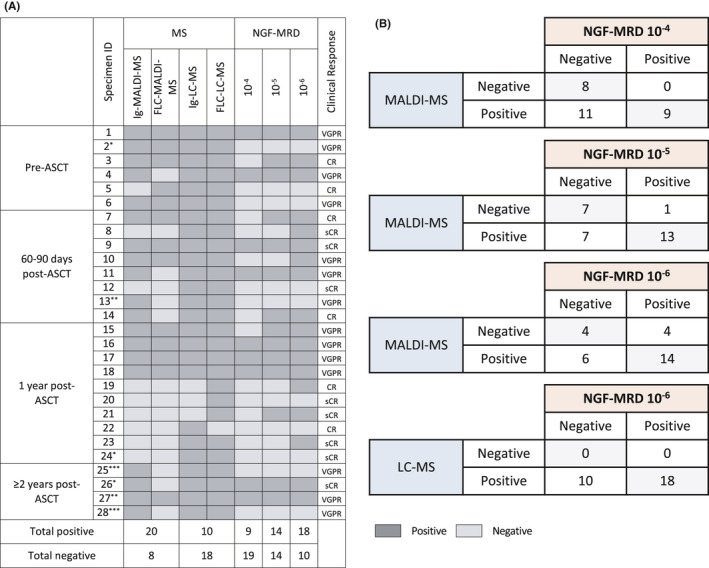
Sensitivity and concordance of MRD testing by MS and NGF. (A). Representation of positive and negative sample results by mass spectrometry and NGF methods at different follow‐up time points. Overall, eight of 28 samples were negative by MALDI‐TOF‐MS. Reflex LC‐MS testing detected intact M‐protein and/or FLC in all eight samples. *, ** and *** indicate serial samples collected from 3 individual patients. (B) Comparative analysis between MS and NGF assessment at different levels of sensitivity

We analyzed 28 paired serum/bone marrow specimens for MRD by MS and NGF. Samples were collected during at least one of the following time points: post‐induction therapy, 60–90 days post‐autologous stem cell transplant (ASCT), 1 or 2 years post‐ASCT. Bone marrow aspirates were prospectively tested using standardized 2‐tubes 8‐colors NGF methods, and MRD status was established at three levels of sensitivity: 10^−4^, 10^−5^, and 10^−6^.^4^ Serum eluates were tested retrospectively by MALDI‐TOF‐MS and Ig‐LC‐MS for the presence of a serum M‐protein using the same *m*/*z* as defined at baseline. All but one patient had an M‐spike <0.2 g/dl, and 14/28 specimens were from patients in ≥CR (including 7 stringent CR). With NGF at 10^−4^, 10^−5^, and 10^−6^ sensitivities, we detected MRD in 9 (32%), 14 (50%), and 18 (64%) samples, respectively (Figure 1A). An M‐protein was identified in 21 (75%) samples by MALDI‐TOF‐MS and in 25 (89%) samples by Ig‐LC‐MS. All specimens positive by MALDI‐TOF‐MS were also positive by Ig‐LC‐MS. FLC‐LC‐MS identified monoclonal FLCs in all but one sample; this sample was positive by Ig‐LC‐MS. Overall, MALDI‐TOF‐MS results were concordant with NGF results for 61% samples at 10^−4^ sensitivity (11 discordant; 11 MS+/NGF−), 71% at 10^−5^ sensitivity (8 discordant; 7 MS+/NGF− and 1 MS−/NGF+), and 64% at 10^−6^ sensitivity (10 discordant; 6 MS+/NGF− and 4 MS−/NGF+) (Figure 1B). LC‐MS detected residual M‐protein and FLC in all specimens, including 10 samples that were negative by NGF at 10^−6^ sensitivity (64% concordance) (Figure 1B). Our results are comparable to MRD concordance rates (NGF and MALDI‐TOF‐MS) published for the GEM‐CESAR high‐risk smoldering MM trial: 81% concordance post‐induction, 70% post‐transplant, and 68% post‐consolidation.^5^ These results are also similar to the 62% concordance rate between MSK 10‐color MRD flow assay (6 × 10^−6^ sensitivity) and MALDI‐MS in patients with MM,^6^ thus highlighting the broad applicability and reproducibility of MS methods in clinical practice.

The value of MS as an ultra‐sensitive serum method for monitoring MRD is illustrated in several cases. For example (Figure S1), a patient with IgAκ MM at first relapse underwent four reinduction cycles (carfilzomib, pomalidomide, and dexamethasone), ASCT, and carfilzomib maintenance. One‐year post‐ASCT, the patient was in stringent CR and MRD negative by both MALDI‐MS and NGF (specimen #24, Figure 1A); however, a small but distinct monoclonal IgAκ peak was detected by Ig‐LC‐MS (MW 23486 Da). Two years post‐ASCT, while in stringent CR, the NGF result was positive for MRD, and both MALDI‐TOF‐MS and Ig‐LC‐MS detected an IgAκ M‐protein with the same molecular mass as at baseline (specimen #26, Figure 1A) demonstrating persistence of the original malignant clone.

In another case (Figure S2), MALDI‐TOF‐MS identified monoclonal glycosylated IgGκ (*m*/*z* 12500–13000) with prominent +2 charge light chain peaks at *m*/*z* 12749 and 12894. This patient underwent five induction cycles (lenalidomide, bortezomib, and dexamethasone), ASCT, and lenalidomide maintenance, achieving a CR. At 1‐year post‐ASCT, NGF results were positive for MRD, but both MALDI‐TOF‐MS and Ig‐LC‐MS showed no M‐protein (specimen #21). Interestingly, FLC‐LC‐MS detected an M‐protein at 25779.1 Da matching the M‐protein mass previously identified. Deglycosylation treatment of the sample demonstrated that the M‐protein produced by the malignant plasma cell clone was indeed glycosylated. This case highlights the increased sensitivity gained by utilizing FLC‐LC‐MS alongside Ig‐LC‐MS, and the potential to use MS for the serial monitoring of glycosylated M‐proteins.

Beyond the discrepancy in M‐protein glycosylation, other discrepancies between NGF and MS results may be related to biology of the disease, since NGF analysis represents marrow‐localized disease, which can be patchy at times, whereas MS‐based analysis signifies more of a secretory disease or extramedullary involvement. Low levels of M‐protein detected by MS‐based assays could potentially suggest a lag between marrow clearance and M‐protein elimination due to the extended half‐life of residual immunoglobulins; however, delayed clearance can be ruled out for several of our patients since MRD was still detected by MS 1 or 2 years after ASCT. From a technical standpoint, hemodilution in bone marrow samples could produce false NGF negative results. We ruled out such interference by conducting mast cell distribution analyses that showed all samples had >0.002% mast cell content (average 0.0104 ± 0.0021% of total nucleated cells). From a clinical standpoint, outcome data are not yet mature for our cohort. After a median follow‐up of 16.2 (range 3.4–33.9) months, the median progression‐free survival was not reached. Of the 12 patients with 2 years of clinical follow‐up post‐ASCT, 6 experienced relapse and 6 remained in remission. While none of the patients who were MRD negative by NGF relapsed, two thirds of patients who were MRD positive by NGF did relapse within this time frame. MALDI‐TOF‐MS identified residual M‐protein/FLC in 11 (92%) patients. Interestingly, LC‐MS identified M‐protein in all those cases, therefore adding little to the biology of the residual disease.

To our knowledge, this is the first report comparing MALDI‐TOF‐MS and LC‐MS serum M‐protein analyses to bone marrow NGF at different sensitivity thresholds. Despite small sample size, our results support including MS analyses in peripheral blood and comparing it with bone marrow‐based MRD in prospective clinical trials of MM. MS analyses, similar to SPEP and IFE, are performed on serum samples, representing a non‐invasive approach. While MALDI‐TOF‐MS is not a quantitative technique, its limit of detection for monoclonal proteins is far lower than conventional M‐protein quantification techniques. It may also allow for the identification of a heavy chain component from some MM patients who are currently diagnosed with FLC MM.

## Supporting information

Fig S1‐S2

## Data Availability

Data can be accessed with permission from the corresponding author.
