# Supplementary material for: Comparison of mass spectrometry and flow cytometry in measuring minimal residual disease in multiple myeloma
Source: Cancer Med. 2021 Sep 8;10(20):6933–6. doi: 10.1002/cam4.4254 (PMC8525140; doi:10.1002/cam4.4254)

Figure S1: Case example demonstrating sensitivity of MALDI-MS and reflex LC-MS. MALDI-MS detected a monoclonal IgAκ (23,478 Da) at first relapse. Pre-transplant (specimen #2), MALDI-MS remained positive although NGF was negative. One-year post-ASCT (specimen #24), MALDI-MS and NGF were negative, however a distinct monoclonal IgA peak was detected by LC-MS (23,486 Da). Two years post-ASCT (specimen #26), NGF was positive and MALDI-MS detected the original IgAκ clone (23,487 Da). Spectra shown for the 2+ charge state of the immunoglobulin light chains.

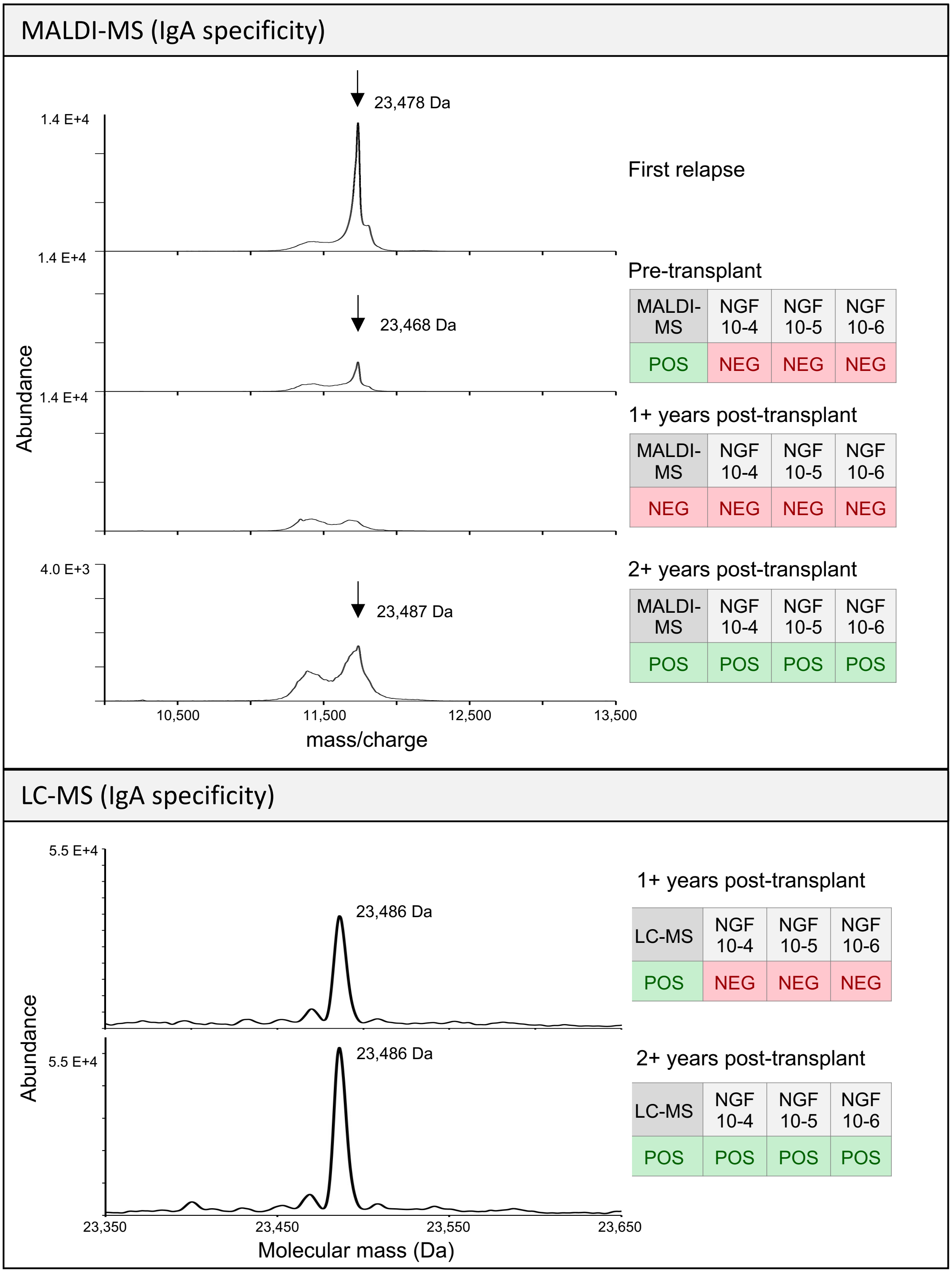

Figure S2: Case example of a glycosylated monoclonal IgGκ. MALDI-MS identified a glycosylated kappa light chain in the m/z 12500-13000 area of the total kappa mass spectra at diagnosis; including prominent peaks at m/z=12749 and m/z=12894 (Figure S2A). One year post-ASCT (specimen #21), MALDI-MS and Ig-LC-MS were negative, however FLC-LC-MS detected the glycosylated light chain (25,778.7 Da; Figure S2B). Spectra shown for the 2+ charge state of the immunoglobulin light chains.

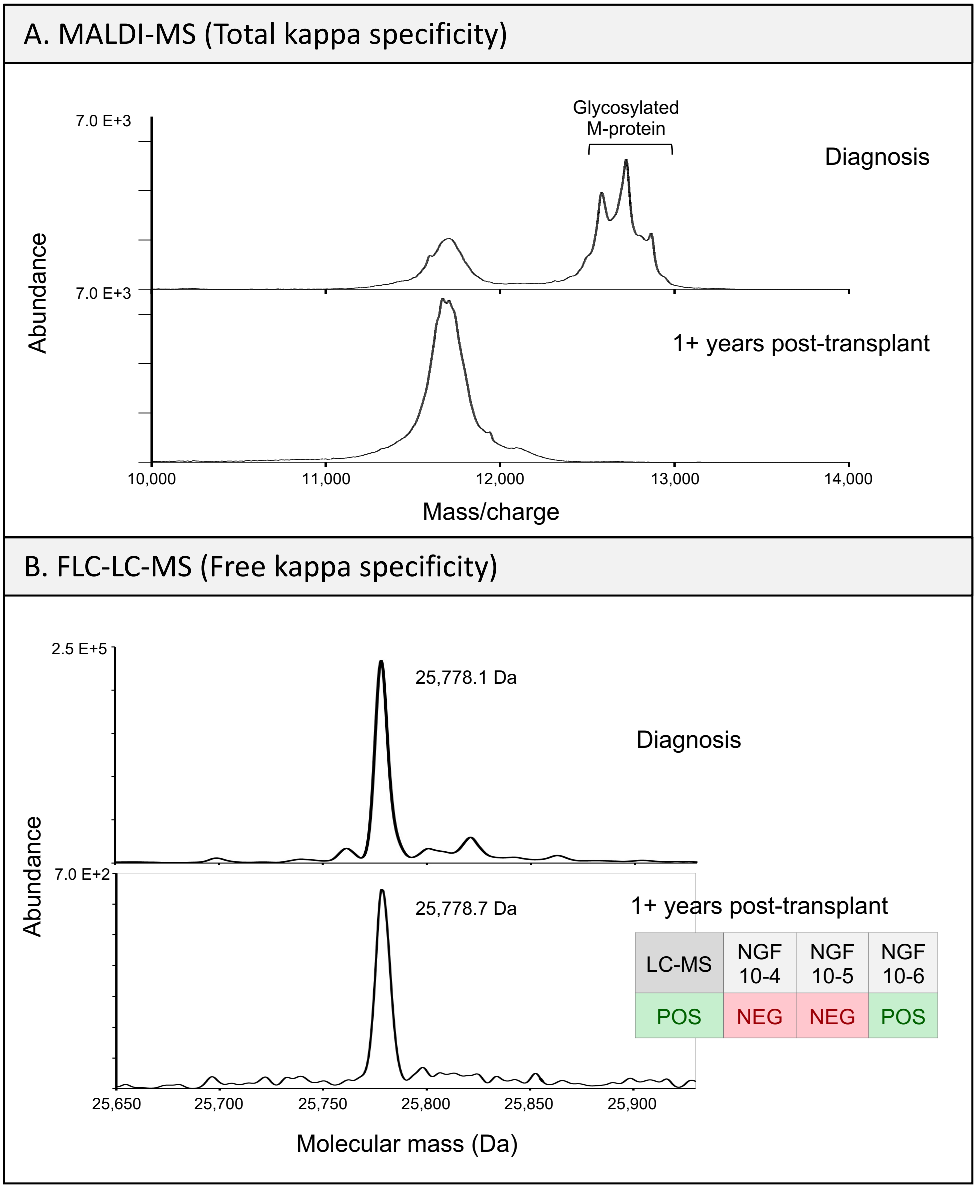

Supplement: Supplementary file 1 — Fig S1‐S2 [file CAM4-10-6933-s001.pdf]
